# A taxonomic, genetic and ecological data resource for the vascular plants of Britain and Ireland

**DOI:** 10.1038/s41597-021-01104-5

**Published:** 2022-01-10

**Authors:** Marie C. Henniges, Robyn F. Powell, Sahr Mian, Clive A. Stace, Kevin J. Walker, Richard J. Gornall, Maarten J. M. Christenhusz, Max R. Brown, Alex D. Twyford, Peter M. Hollingsworth, Laura Jones, Natasha de Vere, Alexandre Antonelli, Andrew R. Leitch, Ilia J. Leitch

**Affiliations:** 1 Royal Botanic Gardens, Kew, Richmond, TW9 3AE UK; 2 School of Biological and Behavioural Sciences, Queen Mary University of London, London, E1 4NS UK; 3 Appletree House, Larters Lane, Middlewood Green, Suffolk, IP14 5HB UK; 4 Botanical Society of Britain and Ireland, Harrogate, HG1 1SS UK; 5 University of Leicester, Leicester, LE1 7RH UK; 6 University of Edinburgh, Edinburgh, EH8 9YL UK; 7 Royal Botanic Garden Edinburgh, Edinburgh, EH3 5NZ UK; 8 National Botanic Garden of Wales, Llanarthne, SA32 8HN UK; 9 Natural History Museum of Denmark, University of Copenhagen, DK-2100 Copenhagen, Denmark; 10 Gothenburg Global Biodiversity Centre, Department of Biological and Environmental Sciences, University of Gothenburg, 405 30 Gothenburg, Sweden; 11 Department of Plant Sciences, University of Oxford, Oxford, OX1 3RB UK

**Keywords:** Plant ecology, Biodiversity, Literature mining

## Abstract

The vascular flora of Britain and Ireland is among the most extensively studied in the world, but the current knowledge base is fragmentary, with taxonomic, ecological and genetic information scattered across different resources. Here we present the first comprehensive data repository of native and alien species optimized for fast and easy online access for ecological, evolutionary and conservation analyses. The inventory is based on the most recent reference flora of Britain and Ireland, with taxon names linked to unique Kew taxon identifiers and DNA barcode data. Our data resource for 3,227 species and 26 traits includes existing and unpublished genome sizes, chromosome numbers and life strategy and life-form assessments, along with existing data on functional traits, species distribution metrics, hybrid propensity, associated biomes, realized niche description, native status and geographic origin of alien species. This resource will facilitate both fundamental and applied research and enhance our understanding of the flora’s composition and temporal changes to inform conservation efforts in the face of ongoing climate change and biodiversity loss.

## Abstract (summary)

**Table:** 

Measurement(s)	Plant Taxonomy • Native status • Functional traits • Ellenberg indicator values • Life strategy • Associated biome • Origin of non-native species • Species distribution • Hybrid propensity • DNA barcodes • Genome size • Chromosome number
Technology Type(s)	digital curation • flow cytometry • Chromosome counts
Sample Characteristic - Organism	Tracheophyta
Sample Characteristic - Environment	archipelago
Sample Characteristic - Location	Great Britain • Ireland

Machine-accessible metadata file describing the reported data: 10.6084/m9.figshare.16451142

## Background & Summary

**Fig. 1:**
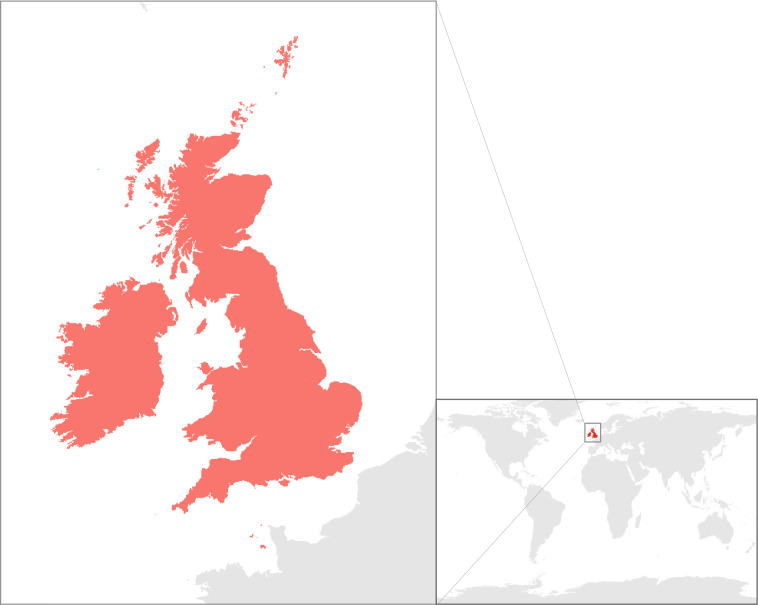
Area covered by the database – Britain and Ireland. The area considered for our attribute database (red) comprises England, Scotland, Wales, Northern Ireland, the Republic of Ireland, the Isle of Man and Channel Islands.

There is a long history of botanical recording on the islands of Britain and Ireland (comprising England, Scotland, Wales, Northern Ireland, Republic of Ireland, Isle of Man and the Channel Islands; Fig. 1, referred to here as ‘BI’), with the earliest systematic records dating back to Sir John Ray in 1690^1^. The Botanical Society of Britain and Ireland (BSBI)^2^ provides access to large-scale geographic distribution data based on more than 40 million occurrence records, allowing for unique research into changes within the flora, especially throughout the last century.

In addition, a large community of researchers have contributed to a wide knowledge base for the BI flora, which includes large datasets on ecological traits, chromosome numbers and cytotype variation, population-level variation and genetic diversity, DNA barcoding resources, and many other traits^3–5^. The conservation status of species in the BI flora has been assessed, including via national red listing^6^. This diversity is protected *in situ* via a range of land management and habitat protection schemes and *ex situ* via large conservation collections and seed banking, with 72% of the UK’s native and archaeophyte angiosperm species (see Online-only Table 1 for a glossary of terms used) currently conserved in seed banks^7^.

BI also have a long history of agricultural development, beginning in prehistoric times^8^ and undergoing a series of changes towards high levels of intensification, especially during the last century^9^. Together these make the region a globally outstanding system for exploring the links between species richness, diverse ecological traits and genetic attributes, allowing for studies on the impacts of environmental and land use change on natural plant communities.

Despite these opportunities, large scale studies of the flora are challenging because of the current lack of a taxonomically-harmonized repository of species present in the BI flora, optimized for comparative flora-wide assessments rather than information retrieval for individual species. The most recent version of a similar data source^10^ dates back to 2004 and almost exclusively covers native species (Online-only Table 1). Another notable inventory, the *List of Vascular Plants of the British Isles*^11^, including both native and alien species, from 1992, has served as the basis for subsequent checklists and keys e.g.^10,12^. Since a large proportion (approx. 50%^13^) of species present in BI today are not native, informed predictions of the species’ future abundance and distribution require that attribute data are readily available for native and alien plants alike. Trait-based approaches to species distribution modelling and community ecology are emerging to enable more informed forecasting of population level responses to changes in the abiotic environment, such as those driven by climate change^14–16^.

Here we present a comprehensive database and inventory of vascular plant species - both native and non-native - currently present in BI, together with diverse trait data. The species list is based on the most recent edition of the *New Flora of the British Isles* (Fourth Edition)^12^ (including name changes from the 2021 reprint), with each species name linked to its unique identification number according to the *World Checklist of Vascular Plants*^17^ to ensure taxonomic clarity and stability.

The repository encompasses 3,209 extant species and 18 species now extinct in BI (see Methods). Each entry includes associated intrinsic and functional traits, distribution and ecologically relevant data where available. In addition to information adapted from Stace (2019)^12^ such as taxonomic ranks, native or alien status and origin (for non-native plants), we have collated other types of data from various sources (Online-only Table 2). These include data for several functional traits (e.g. Specific Leaf Area (SLA), and seed mass), realized niche descriptions (Ellenberg’s indicator values^18^, Online-only Table 1), the life strategy of each species using the CSR strategy framework of Grime (1974)^19^ (Online-only Table 1), information on hybridization propensity, genome sizes and chromosome numbers, along with DNA barcode sequences.

We consider that this comprehensive data repository will be crucial for enabling both fundamental and applied research to enhance our understanding of the biotic and abiotic factors influencing the distribution and composition of the vascular plant flora of BI. Such new insights will be invaluable for predicting how different species will respond to environmental challenges such as biodiversity loss, climate change, land use change and new pests and diseases and hence enable more informed decision making to ensure the long-term stewardship of the BI flora.

## Methods

**Fig. 2:**
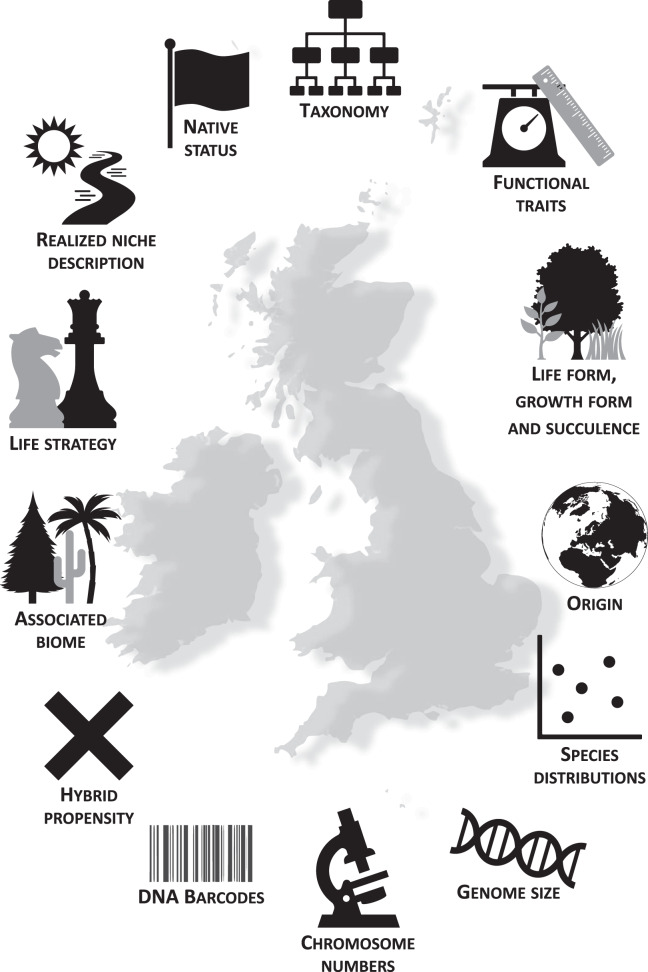
Visualization of the attributes presented in the database.

The broad categories of data included in the repository are summarized in Online-only Table 2 and visualized in Fig. 2. Each category is explained in greater detail below, while full details together with accompanying notes are given in the repository (Database_structure.csv) and in Supplementary File 1. Online-only Table 2 gives an overview of data coverage per category, both across all species and for native species separately. A complete list of data sources is available in Supplementary File 2.

### Generation of the species list

Taxon names listed in the most recent and widely accepted *New Flora of the British Isles’* index^12^ were digitized via the Optical Character Recognition Software Readiris^TM^ 17 (IRIS). Results from the digitization were transferred into a spreadsheet and obvious recognition errors were fixed. The resulting table contained 5,687 taxa and associated taxonomic authorities. A total of 360 unnamed hybrids were excluded, as well as species noted to have only questionable or unconfirmed records, leaving 5,038 species. Forty-one intergeneric hybrid species, 827 entries relating to (notho)subspecies, (notho)varieties, cultivars and *forma* were also removed along with 720 named hybrids. Species that were included by Stace^12^ but which he considered not to be part of the flora (i.e. listed as ‘other species’ and ‘other genera’, e.g. genus *Tragus* or *Coreopsis verticillata*) were also excluded. Seven species that were labelled ‘extinct’ in the flora were included as there were indications that the species might be in the process of reintroduction (e.g. *Bromus interruptus*, *Bupleurum falcatum* and *Schoenoplectus pungens*). Extinct native and archaeophyte species without any signs of reintroduction (e.g. *Dryopteris remota*) are also listed but no additional data are provided and they are not included in calculations of completeness of data (Online-only Table 2). The final number of extant species listed here is therefore 3,209 (comprising 1,468 natives, 1,690 aliens and 51 species with unknown status), plus 18 formally extinct species (natives and archaeophytes not seen in the study region since 1999). Species names and taxonomic authorities were revised according to the 2021 reprint of the *New Flora of the British Isles*, communicated to us by C.A.S. ahead of publication. Genera with less well-defined species – for example due to apomixis – contain additional information on subgenera, sections, and aggregates, as per Stace^12^. Since misidentifications are common in these groups, we include a column termed ‘unclear_species_marker’ that allows for these species to be quickly identified and excluded from analyses if appropriate. Such genera are often incompletely listed in our database since most microspecies are not sufficiently well defined.

### Taxonomy

Nomenclature of the list was checked by Global Names Resolver in the R package ‘taxize’^20,21^, using the *International Plant Names Index* (IPNI)^22^ as the data source, to remove any digitisation errors. Resolved names were used to determine accepted higher taxonomic hierarchy (family, order) again using taxize, with the National Center for Biotechnology Information (NCBI) database. Species that could not be resolved by the Global Names Resolver or did not yield matches in the NCBI database for their higher taxonomic ranks were manually checked for name matches in the *World Checklist of Vascular Plants* (WCVP)^17^. Species within the original species list that were found to be identical to a different spelling in WCVP were retained in the database. In such instances, and when slight spelling differences occurred, the columns ‘taxon_name‘ and ‘taxon_name_WCVP‘ differ. To improve clarity, each species is presented here with its unique identification number according to the WCVP (listed as ‘kew_id’) together with three additional columns (i.e. WCVP.URL, POWO.URL and IPNI.URL) which contain hyperlinks to the freely accessible taxon description websites of the (WCVP)^17^, *Plants of the World Online* (POWO)^23^ and (IPNI)^22^, respectively. Thus, while the taxon names used in the database correspond to those used by Stace^12^, changes in the accepted species name since publication can be traced in columns ‘taxonomic_status’ and ‘accepted_kew_id’. The family classification of WCVP follows APG IV^24^ for angiosperms, Christenhusz *et al*. (2011)^25^ for gymnosperms and Christenhusz & Chase (2014)^26^ for ferns and lycopods.

### Native status

We offer three different datasets which describe the status of a species as native or non-native, and its level of establishment in BI. The first is extracted from Stace (2019)^12^, the second contains the status codes used in PLANTATT^10^ and the unpublished ALIENATT (pers. comm. author K.J.W.) dataset, and the third is extracted from *Alien Plants*^13^. The status from Stace^12^ and Stace & Crawley^13^ assigns a species to either native or alien status, with aliens subdivided into archaeophytes and neophytes at different levels of establishment (e.g. denizen, colonist etc., see Online-only Table 1). Status codes from the BSBI can be either AC (alien casual), AN (neophyte), AR (archaeophyte), N (native), NE (native endemic) or NA (native status doubtful).

### Functional traits

Data for five ecologically relevant functional traits (i.e. seed mass, specific leaf area [SLA], leaf area, leaf dry matter content [LDMC] and vegetative height) were downloaded from public data available in the TRY database^27^ (for specific authors see Supplementary File 1 and Supplementary File 2). Averages were calculated using the available measurements downloaded for each species, excluding rows where the measurement was 0. In addition, the maximum vegetative height for each species is given, where available.

### Realized niche description

Realized niche descriptions based on assessments made on plants living in BI are given in the form of Ellenberg indicator values^18^, as published in PLANTATT^10^. Ellenberg indicator values place each species along an environmental gradient (e.g. light or salinity) by assigning a number on an ordinal scale, depending on the species preference for the specific gradient (Online-only Table 2). This information is often used to gain insights into environmental changes based on species occurrences^28^. For species listed under a previously accepted name in PLANTATT, the information was associated with the accepted synonym in Stace (2019)^12^. Due to the low coverage of PLANTATT for non-native species included in our list, we additionally include Ellenberg indicator values based on Central European assessments, as made available by Döring^29^. Each Ellenberg category is listed in a separate column, keeping the information from both data sources separate to avoid confounding of assessments based on two different regions (i.e. Britain and Ireland versus Central Europe).

### Life strategy

To characterize the life strategy of a species, we used the CSR scheme developed by Grime^19^, which classifies each species as either a competitor (C), stress tolerator (S), ruderal (R) or a combination of these (e.g. CS, SR). CSR classifications were obtained from the *Electronic Comparative Plant Ecology* database^30^. Due to the low coverage of available CSR assessments for species in our database (i.e. data available for just 460 out of 3,209 species) we imputed CSR strategies for a further 981 species using available functional trait data, following the method proposed by Pierce *et al*.^31^. The functional leaf traits required for this method – i.e. specific leaf area, leaf area, leaf dry matter content – were obtained from the TRY database^27^. Pre-existing^30^ and newly imputed CSR strategies are listed in separate columns.

### Growth form, succulence and life-form

Plant growth form descriptions were obtained from the TRY database^27^ and filtered for those entries given by specific contributors (Online-only Table 2) to maintain consistent use of growth form categories. Information on whether a species was considered to be a succulent was obtained by screening the entire growth form information obtained from the TRY database for the phrase ‘succulence’ or ‘succulent’.

Species life-form categories according to Raunkiaer^32^ were determined for each species in our dataset with regard to the typical life-form of the species as it grows in BI (pers. comm. M.J.M.C.).

### Associated biome and origin

Information given in the Ecoflora database^3^ for the biome that each species is associated with was matched to the species names according to Stace^12^. The recognized biome categories follow Preston & Hill^33^ and are ‘Arctic montane’, ‘Boreal Montane’, ‘Boreo-Arctic Montane’, ‘Boreo-Temperate’, ‘Mediterranean’, ‘Mediterranean-Atlantic’, ‘Southern Temperate’, ‘Temperate’, ‘Wide Boreal’ and ‘Wide Temperate’.

For non-native species, the assumed origin (i.e. the region that plants were most likely to have been introduced to BI from, rather than the full non-BI distribution of a species) was adapted from Stace^12^ into a brief description of their country or region of origin. In addition, these descriptions were manually allocated to the TDWG level 1 regions listed in the World Geographical Scheme for Recording Plant Distributions (WGSRPD, TDWG)^34^.

### Species distributions

Distribution metrics for each species are given as the number of 10-km square hectads in BI with records for the species in question within a specified time window. The data were derived from the BSBI Distribution Database^35^ and were extracted for each species, dividing the study region into Great Britain (incl. Isle of Man), Ireland and the Channel Islands, as previously partitioned for data available in PLANTATT^10^. The database was queried using species and hectads for grouping, showing only records ‘matching or within 2 km of county boundary’ and excluding ‘do-not-map-flagged occurrences’. The data were not corrected for sampling bias and should therefore only be used as an indication of trends.

### Hybrid propensity

Data on hybridization is provided for 641 species, obtained from the *Hybrid flora of the British Isles*^36^ which enumerates every hybrid reported in BI up until 2015 (pers. comm. M.R.B.). Each entry was transcribed manually, and then filtered to exclude (a) hybrids that have been recorded, but not formed in the British Isles, (b) triple hybrids (mainly reported for the genus *Salix*), (c) doubtful records, (d) hybrids between subspecific ranks, and (e) hybrids where at least one parent is not native (only archaeophytes included). This left 821 hybrid combinations for data aggregation. The metric chosen here is hybrid propensity, which is a per-species metric of how many other species a focal species hybridizes with (*sensu* Whitney *et al*., 2010^37^). A scaled hybrid propensity metric is also given which was calculated by weighting the hybrid propensity score by the number of intrageneric combinations for a given genus, to account for the greater opportunities of hybridization in larger genera.

### DNA barcodes

DNA barcode sequences for plant species present in BI are currently available for 1,413 species in our database. The information was derived from a dataset of *rbcL*, *matK* and ITS2 sequences compiled for the UK flora generated by the National Botanic Garden of Wales and the Royal Botanic Garden Edinburgh^38,39^ (pers. comm. L.J. and N.D.V.). The data are given as a hyperlink to the record’s page on the Barcode of Life Data Systems (BOLD^40^) which includes the DNA barcode sequences as well as scans of the herbarium specimen and information on the sample’s collection. Most species have multiple record pages associated with them, due to the sampling of more than one individual. We include a maximum of three BOLD accessions per species; the full range of individuals sampled can be accessed via the original publications^38,39^. DNA barcodes are almost exclusively available for native species. Future releases of our database will increase the coverage of the non-native flora significantly. Where species in the BOLD database are attributed to a species name that is considered synonymous with another name in our list, the hyperlink is matched to the latest nomenclature^12^. 1,421 species have at least one sequence associated with them and 935 species have sequence data for all three sequences (*rbcL*, *matK* and ITS2).

### Genome size and chromosome numbers

Genome size data for 2,117 specimens (at least one measurement per species) were obtained from various sources. Measurements for a total of 467 species were newly estimated using plant material of known BI origin, often sourced  from the Millennium Seedbank of the Royal Botanic Gardens, Kew (RBG Kew)^41^. The measurements were made by flow cytometry using seeds or seedlings and following an established protocol^42^. Information on the extraction buffers and calibration standard species used are available in the file GS_Kew_BI.csv, along with peak CV values of the measurements as a quality control. Where more than one measurement is reported per species, the measurements were made on plant material from different populations or using different buffers. Previously published data for additional species were obtained from reports on the Czech flora^43^, the Dutch flora^44^, and prime values listed in the Plant DNA C-values database^45,46^. Since significant intraspecific differences in genome size between plant material from different geographical origins have previously been described, predominantly due to cytotype diversity in ploidy level^47^, genome size measurements from previously published sources were assessed with regard to the origin of the material. The column ‘from_BI_material’ (GS_BI.csv, BI_main.csv) allows users to filter for measurements made on material from BI to exclude a potential bias. The information was obtained from the original publication source of each measurement.

Chromosome numbers for 1,410 species (at least one chromosome number per species) determined exclusively from material collected in BI were obtained from an extensive dataset compiled by R.J.G. from various published studies, unpublished theses and personal communications from trusted sources. The counts were made between 1898 and 2017, with a large proportion stemming from efforts to achieve greater coverage of the flora by a team of cytologists based at the University of Leicester and headed by R.J.G. Part of the dataset was previously incorporated into the BSBI’s data catalogue^5^ but has since undergone revisions to incorporate new information and changes in taxonomy. The dataset contained many measurements at subspecies level which were allocated to the species level taxon in our list. This served to include as much of the often considerable infraspecific variation as possible. Since some species for which chromosome counts have been reported elsewhere are lacking chromosome counts from British or Irish material, they are absent from this dataset. To fill such gaps, we also present chromosome numbers from reports on the Czech flora^43^, the Dutch flora^44^, and the Plant DNA C-values database^45,46^.

## Data Records

A static version of the data as of publication date is available from the NERC Environmental Information Data Centre (10.5285/9f097d82-7560-4ed2-af13-604a9110cf6d)^48^. A metadata file (Database_structure.csv) with explanations of the main dataset (BI_main.csv), additional datasets (GS_BI.csv, GS_Kew_BI.csv and chrom_num_BI.csv), and a complete list of all publications and sources used to compile the data (Detailed_sources.csv) are included along with the data. The main database BI_main.csv lists all taxa included in this work along with their identification number (kew_id), associated taxonomic authorities, taxonomic ranks (order, family, genus, subgenus, section, subsection, series, species, group, aggregate), associated trait, distribution, and ecological data. The main database contains a summary of chromosome numbers and the smallest genome size measurement available per species.

Because more than one chromosome number and genome size measurement has been reported for many species – often reflecting considerable infraspecific variance – these additional chromosome number (chrom_num_BI.csv) and genome size (GS_BI.csv) data are published along with the main dataset as separate files. Detailed information about the newly generated genome size measurements from RBG Kew are summarized in GS_Kew_BI.csv, including information on the calibration standard species and extraction buffers used to estimate the genome size.

The data is also available as an R package on GitHub (https://github.com/RBGKew/BIFloraExplorer^49^) where we aim to provide new releases regularly that will reflect new additions to the dataset as well as taxonomic changes.

## Technical Validation

The data were compiled from a range of sources, the vast majority of which were from previously published field guides, atlases or peer reviewed articles. All such data are provided with full reference to their source (see Supplementary File 1 and Supplementary File 2), allowing the user to validate particular pieces of information with ease. Any new unpublished data presented here were either determined experimentally, following best practice protocols (i.e. genome size data), calculated using peer reviewed methods^31^, or supplied by one of the expert authors on this publication.

Where data were manually extracted from print sources, spot checks were conducted at various stages throughout the data collection to verify that mistakes had been kept to a minimum. When data were added from online or other digital resources, species binomial and – if available – taxonomic authority information were used to match data to the species in the list. This matching process was manually checked for each dataset.

## Usage Notes

We present an easily accessible and downloadable database for the current vascular flora of Britain and Ireland, comprising a full list of species with a range of associated ecological, genomic and distribution data. The data as of publication date are freely available for download from the EIDC (10.5285/9f097d82-7560-4ed2-af13-604a9110cf6d)^48^. Species names are presented as published previously^12^ (with name changes from the 2021 reprint); changes in taxonomy are reflected in columns ‘accepted_kew_id’, ‘accepted_name’ and ‘accepted_authors’, as per WCVP and POWO. The development version of the dataset is available at https://github.com/RBGKew/BIFloraExplorer^49^.

## Supplementary information

Supplementary File 1

Supplementary File 2

## Data Availability

**Feedback, community engagement and updates** The R data package presented on GitHub (https://github.com/RBGKew/BIFloraExplorer)^49^ is intended to be a dynamic representation of the data. As changes in the flora arise or new associated information becomes available, these will be incorporated into future releases of the R package, allowing for a dynamic representation of the changing flora as well as version control reflecting database development. While there are gaps in our current knowledge, especially regarding non-native species in Britain and Ireland, we aim to update the dataset as new information becomes available. The data stored in the EIDC repository will remain static and reflect the dataset as of publication date of this data descriptor.

## Online-only Tables

**Online-only Table 1:** Glossary of terms used in the data descriptor and repository.

Category	Term	Description
Native status	Native	Species which colonized the study region naturally since the last glaciation or that was present before that point
	Alien/ Non-native	Species which were most likely introduced by human activity, they are further subdivided into archaeophytes and neophytes
	Archaeophyte	Non-native that was introduced by human activity before the year 1500
	- Colonist	- Weedy species occurring on open ground
	- Cultivated	- Deliberately cultivated species
	- Denizen	- Species with near-native behavior, able to compete with natives
	Neophyte	Non-native that was introduced by human activity since the year 1500
	- Casual	- Not naturalized, persists only for a short time
	- Naturalized	- Established and self-perpetuating
	- Survivor	- Not naturalized, but able to persist for long times, often as a relic in location where it was planted
	Neonative	Species that arose from natural hybridization between either a native and an alien or between two alien taxa, or that evolved from another neonative or alien species within Britain & Ireland
Genome size	Genome size	The amount of DNA in an unreplicated nucleus as estimated by flow cytometry, given as 1 C (haploid nucleus) and 2 C (diploid nucleus), measured in picograms (pg) or mega base pairs (Mbp)
Realised niche	Ellenberg indicator values	Ordinal data for the preference of a species within an environmental gradient; data given for light, moisture, soil acidity, soil fertility, salt and temperature (each species is assigned a value (typically from 1 to 9) depending on its predicted preference within the environmental gradient); concept developed by Ellenberg^18^
Life strategy	CSR strategy	Functional classification of each species’ propensity for being a competitor (C), stress-tolerator (S) or ruderal (R); developed by Grime^19^
Life-form *sensu* Raunkiaer^32^	Hydrophyte	Aquatic herb, buds are submerged in water or in soil underneath water, leaves may float or be submerged, flowering parts may emerge (=‘aquatics’)
	Helophyte	Buds are fully submerged in water or within water-saturated soil, flowers and leaves emerge fully (=‘emergents’)
	Geophyte	Above ground parts die outside the growing season, plant survives as a bulb, rhizome, tuber or root bud
	Hemicryptophyte	Herbaceous stems that tend to die back outside the growing season, buds survive on or just under the soil level, includes many biennial and perennial herbs
	Therophyte	Life cycle is completed within one growing season, surviving as a seed until the next growing season (=‘annuals’)
	Chamaephyte	Herbaceous or woody stems, buds above soil, but not exceeding 50 cm (=‘shrubs’)
	Phanerophyte	Persistent, woody stems, buds usually 3 m or more above ground, trees and larger shrubs (=‘trees’)

**Online-only Table 2:** Summary of the categories included in the database of vascular plants in Britain and Ireland.

Category	Percentage of species with data in the complete flora (percentage for natives/ non-natives given in brackets)	Databases and other reference sources of the data	Description
Taxonomy	100% (*100%/100%*)	Nomenclature and lower taxonomic ranks – Stace (2019, reprint 2021); World Checklist of Vascular Plants (WCVP) Higher taxonomic ranks (order, family) – NCBI via ‘taxize’, WCVP	Overview of species taxonomy, including kew_id, sp ecies binomials (Stace, 2019 (reprint 2021); WCVP), taxonomic rank (i.e. order, family, genus, subgenus, section, subsection, series, species, group, aggregate). Also provided are URLs to species pages on WCVP, POWO and IPNI.
Native status	(i) 98% (*−/−*)	(i) Stace (2019)	Description of level of nativity or establishment in Britain and Ireland (‘Native’, ‘Archaeophyte denizen’, ‘Neophyte naturalized’ etc., for full list see Supplemental Table 1)
	(ii) 82% *(−/−)*	(ii) PLANTATT (Hill *et al*. 2004) and ALIENATT (pers. comm. K.J.W.)	
	(iii) 48% *(−/−)*	(iii) *Alien Plants* (Stace & Crawley, 2015); pers. comm. K.J.W.	
	Combined coverage: 99%		
Functional traits	SLA: 56% *(69%/45%)*	Public data from the TRY database (Kattge *et al*., 2020); for a list of specific publications (see Supplemental Table 2)	Functional plant trait averages for (i) Specific Leaf Area (SLA, mm^2^ mg^−1^), (ii) Leaf Dry Matter Content (LDMC, g g^−1^), (iii) Seed mass (mg), (iv) Leaf area (mm^2^), and (v) Vegetative height (m). Also included is maximum vegetative height (m)
	LDMC: 47% *(65%/32%)*		
	Seed mass: 68% *(74%/63%)*		
	Leaf area: 51% *(66%/39%)*		
	Vegetative height: 75% *(88%/65%)*		
Realized niche description	Percentages given for each Ellenberg category, first the coverage derived from PLANTATT, then from Döring, 2017, then coverage for both sets combined:	(i) PLANTATT (Hill *et al*. 2004) (ii) Zeigerwerte von Pflanzen & Flechten in Mitteleuropa (Döring, 2017)	Ellenberg indicator values assigned to plant species as observed in Britain (data from PLANTATT) and in Central Europe (data from Döring, 2017). Listed Ellenberg categories are L (light), F (moisture, from German ‘Feuchtigkeit’), R (reaction, soil acidity), N (nutrients, fertility), S (salt), T (temperature, only for European data). Numbers typically range across a scale of 1 to 9, with low numbers indicating an affinity to the lower end of the described environmental gradient. S and F have different scales with S spanning from 0 to 9 and F spanning from 1 to 12.
	L: (i) 56% *(94%/23%)*		
	(ii) 60% *(94%/32%)*		
	61%		
	F: (i) 56% *(94%/23%)*		
	(ii) 59% *(92%/31%)*		
	61%		
	R: (i) 56% *(94%/23%)*		
	(ii) 55% *(87%/29%)*		
	60%		
	N: (i) 56% *(94%/23%)*		
	(ii) 58% *(91%/30%)*		
	60%		
	S: (i) 56% *(94%/23%)*		
	(ii) 61% *(95%/32%)*		
	61%		
	T: (i) - *(−/−)*		
	(ii) 27% *(38%/17%)*		
	—		
Life strategy	(i) 14% *(27%/4%)*	(i) Electronic Comparative Plant Ecology (Hodgson *et al*., 1995)	Life strategy of plants given as the CSR category established by Grime (1974). These can be either competitor (C), stress tolerator (S), ruderal (R), or a combination of these (e.g. CS, C/CSR)
	(ii) 45% *(63%/30%)*	(ii) Inferred from functional traits	
	Combined coverage: 45%		
Growth form and succulence	(i) 86% *(89%/83%)* for growth form	Public data from the TRY database (Kattge *et al*., 2020), for specific references, see Supplemental Table 2	(i) Plant growth form given as recorded by the TRY contributors Engemann and Günther. Categories used are aquatic, fern, graminoid, herb, shrub, and tree.
	(ii) 16 succulent species		(ii) Succulence was recorded when a species was mentioned as ‘succulent’ by any author in the growth form data from the TRY database (16 species).
Life-form	100% *(100%/100%)*	Pers. comm. M.J.M.C.	Life form categories as per Raunkiaer (1934) (e.g. ‘chamaephyte’, ‘hemicryptophyte’, ‘therophyte’ or combinations thereof, see Table 1 for explanations)
Associated biome	48% *(86%/15%)*	Ecoflora database (Fitter & Peat, 1994)	Description of typical biome for the species (e.g. ‘Mediterranean’ or ‘Boreo-Temperate’)
Origin of non-native species	(i) 48% *(-/87%)*	Stace, 2019	(i) Description of country or region of origin (i.e. the most likely area plants were introduced from; not equal to complete foreign distribution) for non-native species.
	*(ii) 46% (-/84%)*		(ii) Information is also given as a TDWG level 1 code (Brummitt, 2001).
Species distributions	98% *(98%/97%)*	BSBI distribution database	Species occurrences within Britain and Ireland at hectad resolution for four time intervals: 1987–1999, post 2000, 2000–2009, 2010–2019.
			Data are given separately for Great Britain and the Isle of Man, Ireland and the Channel Islands.
Hybrid propensity	20% *(30%/11%)*	Stace *et al*., 2015; pers. comm. M.R.B.	Hybrid propensity (*sensu* Whitney *et al*., 2010), scaled hybrid propensity (weighted by the number of intragenic combinations within the genus)
DNA barcodes	44% *(87%/11%)* (with at least one record on BOLD), 935 species have sequence data for all three sequences (*rbcL, matK* and ITS2)	Pers. comm. L.J. & N.D.V., de Vere *et al*., 2012, Jones *et al*., 2021	Hyperlinks to the Barcode of Life Data System (BOLD) record pages, which contains barcode sequences (*rbcL*, *matK* and ITS2), an image of the scanned herbarium specimen and details about sample collection
Genome size	66% *(77%/58%)* (with at least one measurement)	(i) Unpublished data from the Royal Botanic Gardens, Kew (RBG Kew) (ii) Šmarda *et al*., 2019 (iii) Zonneveld, 2019 (iv) Plant DNA C-values database (Pellicer & Leitch, 2020)	Genome size measurements, given as 1C- and 2C-values in picograms (pg) and megabase pairs (Mbp)
	14% *(27%/4%)* (with at least one measurement from material sourced from the study region)		
Chromosome numbers	44% *(76%/17%)* (with at least one measurement from material sourced from the study region)	Database curated at the University of Leicester by R.J.G.	Chromosome counts and estimates prepared from plant material from Britain and Ireland, an additional column adds further chromosome numbers from outside of the study area
	72% (*91%/57%*) (with chromosome numbers available from all sources combined)	(i) Database curated at the University of Leicester by R.J.G. (ii) Šmarda *et al*., 2019 (iii) Zonneveld, 2019 (iv) Plant DNA C-values database (Pellicer & Leitch, 2020)	
